# 

**Published:** 1890-02

**Authors:** 


### CONTENTS:

Article I.—Looking Forward. - - - - - - 433

" II.—Pyorrhœa Alveolaris. - - - - - - - 444

" III.—Aluminum as a Base for Artificial Dentures. By A. Hugh Hipple, D. D. S. - - - - 450

" IV.—Dental Education. By S. J. Andres, L. D. S. - - 456

" V.—Dental Caries. By Henry Sewill, M.R.C.S. and L.D.S. 461

" VI.—Excessive Hemorrhage after Extraction of Teeth—A Case. By C. H. M. Neall, M.D., D.D.S. - 464

" VII.—Address Delivered before the Maryland State Dental Association, February 28th, 1889. By Wm. A. Mills, D. D. S. - - - - - 467

### COMMUNICATIONS

American Academy of Dental Science. - - - - - 470

Some Further Notes on the Invention of Porcelain Teeth by the Late Chas. W. Peale. - - - - - - 473

### EDITORIAL.

University of Maryland Dental Department. - - - - 475

The Amendments to the Maryland State Dental Law. - - 477

### BIBLIOGRAPHICAL.

Artificial Crown and Bridge-work. - - - - - - 478

Dental Anatomy—Human and Comparative. - - - - 478

Transactions of the American Dental Association. - - 479

Artificial Anæstheisa. - - - - - - - 479

Practical Electricity in Medicine and Surgery. - - - 480

